# The role of physical exercise in promoting bystander intervention in school bullying: evidence from cross-sectional and longitudinal studies

**DOI:** 10.3389/fpsyg.2026.1718104

**Published:** 2026-05-08

**Authors:** Xinwei Zhou, Yanlan Chen, Hui Xiao

**Affiliations:** 1 School of Physical Education and Health, Jiangxi Science and Technology Normal University, Nanchang, Jiangxi, China; 2 School of Physical Education, Guangdong University of Education, Guangzhou, Guangdong, China

**Keywords:** bystander intervention behavior, embodied cognition, physical exercise, physical self-efficacy, sense of justice

## Abstract

**Background:**

School bullying is a pervasive global concern that profoundly affects teenagers’ development and mental health. This study examines the influence of physical exercise on bystander intervention in bullying, analyzing the psychological mechanisms that underpin this relationship through the frameworks of embodied cognition and mind–body interaction theories.

**Objective:**

The study aims to determine if physical exercise facilitates bystander intervention in school bullying by improving physical self-efficacy and a sense of justice.

**Methods:**

In 2024, a cross-sectional survey was administered with 954 middle school students (566 males, 388 females; mean age 13.85 years) in Jiangxi, China. Participants completed validated instruments measuring physical exercise, self-efficacy, sense of justice, and bystander intervention behavior. Data were analyzed utilizing SPSS and AMOS structural equation modeling (SEM) to evaluate the correlation between physical exercise and bystander conduct, along with the mediating effects of physical self-efficacy and sense of justice. A duration of 8 weeks involving martial arts with 51 male pupils was employed to investigate potential causal links. A Repeated-Measures ANOVA was performed to assess the impact of the intervention.

**Results:**

Structural equation modeling indicated that physical exercise strongly predicts bystander intervention behavior (*β* = 0.124, *p* < 0.01), with physical self-efficacy (*β* = 0. 118, *p* < 0.01) and sense of justice (*β* = 0.095, *p* < 0.01) acting as mediators. The intervention outcomes indicated enhancements in physical self-efficacy and sense of justice, resulting in a rise in bystander intervention.

**Conclusion:**

These findings indicate that physical exercise can significantly facilitate the transition of bystanders from passive viewers to proactive helpers, providing essential insights for the mitigation and prevention of school bullying.

## 1 Introduction

Bullying in schools is a worldwide problem with detrimental physical and psychological consequences for the adolescents. In China, more than half of the students have been victims of bullying (Huang, 2024) and therefore understanding of factors affecting their responses is essential (Aisyah et al., 2025).

Most studies have been about bullies and victims; limited attention has been paid to bystanders, who have the potential to influence the course and outcome of bullying (Zhang and Dong, 2019; Jing et al., 2005). Bystanders’ behaviors are closely connected with the dynamics of bullying, and interventions can mitigate their negative consequences (Wu et al., 2022; Stewart, 2020). Nonetheless, many students are reluctant to take action because of low levels of self-efficacy, moral disengagement, or peer influence, while empathetic students or those in supportive classrooms are more likely to take action (Jing et al., 2005; Yang and Wang, 2012; Fu and Ding, 2024; Wu et al., 2022; Stewart, 2020; Thornberg and Jungert, 2013; Zhang and Risen, 2014).

A bystander is classified as a person who is a witness of bullying and is not involved directly as a perpetrator or victim (Latane, 1970). The bystander effect is defined as the decreased probability of intervention when others are around as a result of the diffusion of responsibility and social influence (Darley and Latane, 1968).

Physical exercise may influence the bystander behaviors among adolescents through physical self-efficacy and moral reasoning. Regular activity increases control of the body and builds self-assurance, which increases readiness for social interactions (Zhang et al., 2015; Wang et al., 2022; Dong et al., 2023; Chen et al., 2020). It also provides embodied experiences of fairness, cooperation, and rule-following, which help develop a sense of justice and strengthen moral judgment, thereby promoting proactive interventionv. (Ludwiczak and Bronikowska, 2022; Ju, 2013; Mahmoud et al., 2022; Gibson, 2014; Zhao, 2019).

Although links between exercise, self-efficacy, and moral development are established, their impact on bystander intervention is less clear. This study combines a cross-sectional survey with an 8-week physical activity intervention to examine both correlational relationships and preliminary causal effects, providing insights into the mechanisms through which exercise may promote active bystander responses in school bullying.

## 2 Theory and hypotheses

### 2.1 Bystander intervention model

This paper will apply the bystander intervention model, developed by Latane (1970) and later revised by Nickerson et al. (2014), to explore the decision-making process of the bystanders. The concept outlines the stages of intervention (that are done in succession): steps of intervention include (1) noticing the scenario, (2) making the situation a scenario that requires help, (3) taking charge, (4) evaluating one’s ability to intervene, and (5) deciding whether or not to act.

Emergency aid is not immediate, and it involves a process of thinking that encompasses focusing on the surroundings, which may ultimately lead to the decision to take action. Social and cognitive variables can hinder the effectiveness of treatment in all stages. Some of the common roadblocks include lack of responsibility dispersion, pluralistic ignorance, and low levels of self-efficacy (Latané and Darley, 1969; Salmivalli et al., 2011). In a bullying scenario, just as it occurs in schools, the bystander effect implies that individuals can intervene as a group because of the presence of others and that people tend to expect others to take action (Darley and Latané, 1968). This model can be used to understand the reasons behind students’ failure to take action once they are aware of the adverse effects of bullying.

### 2.2 Embodied cognition theory and physical exercise

Physical factors can influence the behavior of bystanders, in addition to cognitive and social processes. The embodied cognition theory suggests that cognition and action are a dynamic process between the body and the surroundings (Wilson, 2002; Glenberg, 2010; Lakoff and Johnson, 1980; Haosheng, 2010). Bodily experiences gained during physical activity can be utilized to create cognitive schemas that influence the degree of self-regulation and shape an individual’s behavior (Shapiro, 2019).

Exercise provides repetitive body reactions that enhance the body’s control, confidence, and psychological strength, which, in turn, can influence intervention behavior (Ye, 2014; Zhang et al., 2015; Guo et al., 2024; Chunxian and Yunliang, 2019). In particular, the adolescents will be able to gain physical self-efficacy, meaning their ability to perform in unfavorable circumstances. Physical self-efficacy is also positively related to proactive behaviors, such as interfering in bullying situations (Wang et al., 2022).

Justice sensitivity is also promoted by exercise, as organized sports and physical activity require following regulations, interacting with others, and promoting equality, which facilitates the internalization of ethical standards (Huang, 2024; Yin and Zhang, 2018). Participation in sports activities of adequate magnitude encourages conformity to regulations and collaboration, which translates abstract laws of justice into mental and emotional understanding, and thus, promotes proactive intervention in bullying scenarios. More sensitive adolescents will act more by labeling bullying a moral matter and discriminating against it (Ju, 2013; Ludwiczak and Bronikowska, 2022; Xia and Ling, 2017; Xiao et al., 2014).

### 2.3 Linking theory to research focus

Together, these cognitive, social, and embodied mechanisms suggest pathways through which physical exercise may promote bystander intervention in school bullying. According to this theoretical framework, the physical self-efficacy and the justice sensitivity are determined to be roles played by both physical exercise and bystander intervention.

**Figure 1:**
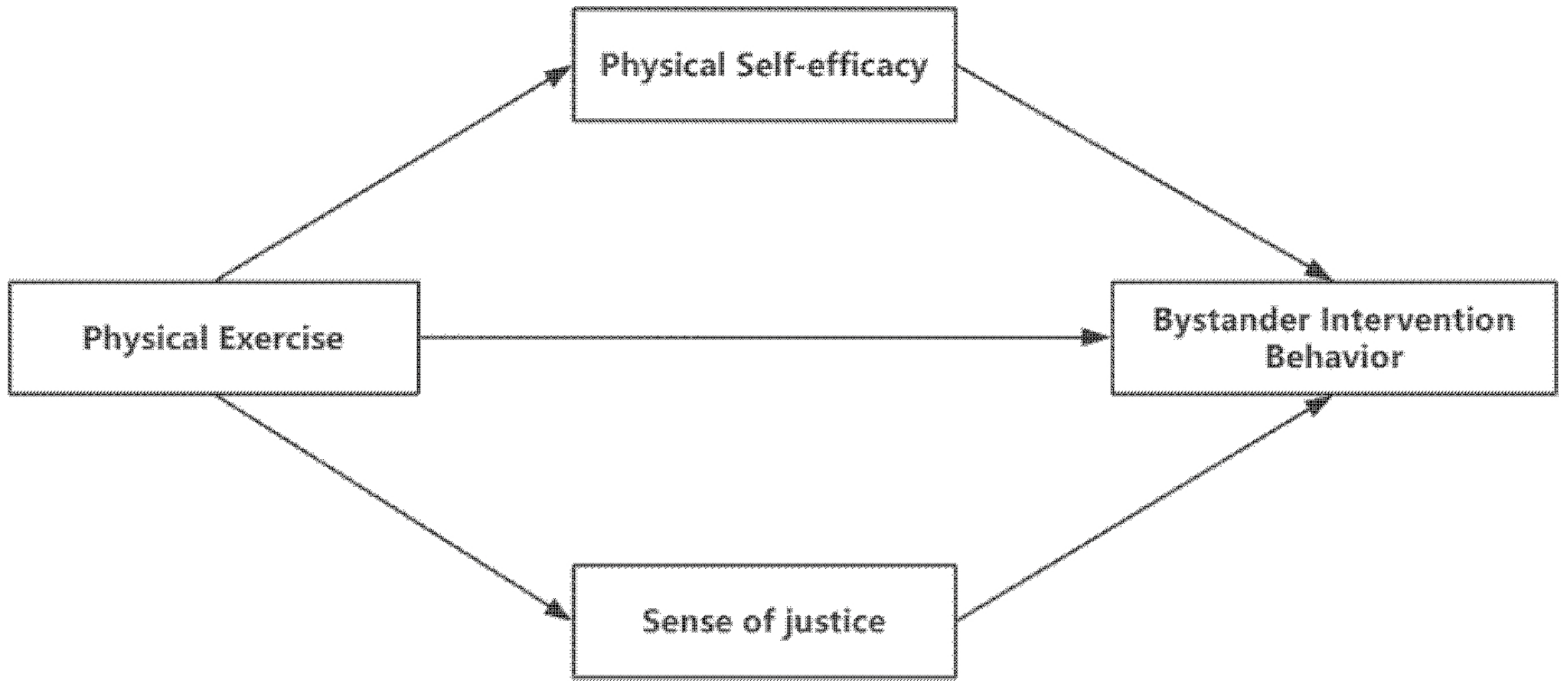
Research hypothesis model diagram.

Drawing on the preceding analysis and theoretical foundations, this study proposes the following hypotheses and model diagram (Figure 1):

*H1*: Physical exercise is a significant positive predictor of bystander intervention in school bullying.

*H2*: Physical self-efficacy mediates the relationship between physical exercise and bystander intervention in school bullying.

*H3*: Sense of justice mediates the relationship between physical exercise and bystander intervention in school bullying.

## 3 Subjects and methods

This study comprises two complementary studies: a cross-sectional survey and an 8-week intervention study using a mixed design.

### 3.1 Subjects

Stratified random sampling was used in this study to identify 991 Grades 7–9 students in four secondary schools in Jiangxi Province. The students were stratified according to grade level and school level. Within each school, the sample was proportionally representative of the grades in that school. The questionnaire was distributed in the classrooms via QR codes, and students were informed about the purpose of the research and the ethical principles. Although QR codes were used, there may have been a slight impact of self-selection and peer-influence problems on the responses.

Cases with excessive missing data or homogeneous answers were filtered out, resulting in a valid response rate of 954 (96.3%). The sample included 566 boys (59.3%) and 388 girls (40.7%). The average age of the respondents was 13.85 years with a standard deviation of 1.39 years. The age distribution was as follows: 12 years (26.9%), 13 years (11.7%), 14 years (22.4%), 15 years (26.9%), and 16 years (11.9%).

The grade level distribution was the following: 318 seventh graders (33.3%), 377 eighth graders (39.5%), and 259 ninth graders (27.1%). The independent variables were gender, age, grade level, whether the individual was an only child, and the type of school attended.

### 3.2 Research tools

#### 3.2.1 Physical exercise rating scale (PARS-3)

The assessment of physical activity was conducted using the Physical Exercise Rating Scale, which Hashimoto Kunio developed and was later revised by Liang Deqing and others in 1994. The scale measures three aspects of exercise, namely intensity, length of the exercise, and frequency. The cumulative score on physical exercise is calculated as follows:

Physical Exercise Score=Intensity(Duration−1)Frequency

The evaluation of each dimension is made with the help of a Likert scale (the highest level is 5 points, and the lowest is 1 point), with the basis of this rating ranging from 0 to 100. As per previous surveys, the scores are categorized into low scores (≤19), moderate scores (20–42), and high scores (≥43) (Yu, 2023). The internal consistency of the current sample was high (Cronbach’s *α* = 0.820), and it has been widely used in Chinese research in the field of public health and physical education.

#### 3.2.2 Physical self-efficacy scale

Physical self-efficacy was measured using the Physical Self-Efficacy Scale (Ryckman et al., 1982; Sun et al., 2005). It is a scale that has been widely applied to adolescents in China. Respondents rated each topic on a 6-item Likert scale, ranging from “strongly disagree” to “strongly agree,” with higher ratings indicating increased physical self-efficacy. The scale was reported to have a reasonable degree of reliability and construct validity in this research (KMO = 0.899, Cronbach’s *α* = 0.863). No internally created items were added.

#### 3.2.3 Sense of justice scale

The Justice Sensitivity Questionnaire (Schmitt et al., 2010; Yang, 2013) was employed to assess sensitivity to injustice from the bystander viewpoint, particularly the observer dimension. The complete JSQ comprises four subscales: victim, observer, beneficiary, and perpetrator, each containing 10 items assessed by a 6-point Likert scale from “strongly disagree” to “strongly agree.”

The observer subscale (Items 11–20) was included only since this research focused on the bystander behavior in bullying at school. High scores indicate greater sensitivity to feelings of unfairness. Observer sensitivity refers to both emotional and moral responses to witnessing injustice. It is directly related to the viewing tendencies. The remaining subscales are not paramount in this study.

The subscale was construct valid and had high reliability (KMO = 0.944; Cronbach’s *α* = 0.896). The JSQ studies have been validated, and no new items have been developed.

#### 3.2.4 School bullying bystander intervention behavior scale

The level of reliability and validity of the Bystander Intervention Scale proved to be high regarding Chinese middle school students (You et al., 2020; Nickerson et al., 2014). The scale was rated based on the bystander’s performance at every stage of intervention. The intervention behavior consisted of five stages: noticing, interpreting, responsibility assumption, knowledge acquisition, and action, totaling 16 items. The responses are rated using a scale from ‘strongly disagree’ to ‘strongly agree’. The scale was highly structurally valid (KMO = 0.838), and the levels of reliability were high (Cronbach’s alpha of 0.707 to 0.825 across all dimensions, with a total alpha of 0.826). Each scale had a factor structure that was consistent with its nature dimensions, thus supporting the validity of the instruments. There was nothing in the form of internally designed goods.

In this study, the Ethics Committee provided its consent, and the research was conducted in accordance with the principles outlined in the Declaration of Helsinki. All participants and their legal guardians were informed about the participation and gave informed consent.

## 4 Results

### 4.1 Study 1: cross-sectional study

#### 4.1.1 Common method bias test

Self-report measures were used to assess all four primary constructs in this study, which put them at risk of common method bias. To solve this, two steps were followed. To begin with, the single-factor test by Harman was conducted through an unrotated exploratory factor analysis of all items. The eigenvalues of the eight factors exceeded one, and the first factor explained 24.97% of the variance, which is substantially smaller than 40%. The size of standard method bias has been minimized. Second, confirmatory factor analysis (CFA) was conducted using AMOS 28.0, where one-factor, two-factor, three-factor, and four-factor models were compared. The four-factor model demonstrated the most adequate fit and high levels of discriminant validity (*x*^2^/*df* = 2.845, CFI = 0.922, IFI = 0.923, RMSEA = 0.044), which also indicated that common method bias was within reasonable levels.

However, it is also notable that all the constructs were self-reported, which could create the possibility of bias related to shared method variance. The limitation could be overcome in future research through the use of multi-source or behavioral measurements.

#### 4.1.2 Descriptive and correlational analyzes of the principal variables

Table 1 represents the descriptive statistics of the main variables. The variables exhibited good variability and were close to a normal distribution. The physical exercise distribution of scores showed moderate positive skewness (0.757) and near-zero kurtosis (−0.066). Due to the large sample size (*N* = 954), parametric analyzes were employed; therefore, bootstrap techniques were used to enhance the strength.

**Table 1:** Descriptive statistics for each variable.

Name	Sample size	Minimum	Maximum	Mean	Standard deviation
Physical exercise	954	0.000	100.000	37.864	26.610
Physical self-efficacy	954	17.000	50.000	39.164	6.891
Sense of justice	954	13.000	50.000	37.771	7.533
Bystander intervention behavior	954	35.000	80.000	62.404	8.972

Analysis of the Pearson correlation (Table 2) revealed substantial and positive correlations among all the main variables. High degrees of physical activity were positively related to increased physical self-efficacy, increased justice sensitivity, and increased bystander intervention behavior. Such descriptive and correlational findings provide a robust statistical basis to test new models in the future and offer preliminary empirical support for the hypotheses.

**Table 2:** Descriptive statistics and correlation analysis of main variables.

Variable	*M*	SD	(1)	(2)	(3)	(4)	(5)	(6)	(7)	(8)	(9)
Physical exercise (1)	37.864	26.610	1								
Physical self-efficacy (2)	38.777	7.494	0.387^**^	1							
Sense of justice (3)	37.308	8.269	0.285^**^	0.346^**^	1						
Intervention behavior (4)	62.178	9.322	0.340^**^	0.480^**^	0.489^**^	1					
Attention (5)	11.131	3.168	0.100^**^	0.121^**^	0.206^**^	0.609^**^	1				
Interpretation (6)	11.227	3.097	9.120^**^	0.125^**^	0.258^**^	0.634^**^	0.476^**^	1			
Responsibility (7)	11.945	2.520	0.305^**^	0.460^**^	0.348^**^	0.677^**^	0.170^**^	0.235^**^	1		
Know (8)	11.980	2.389	0.224^**^	0.304^**^	0.390^**^	0.610^**^	0.191^**^	0.220^**^	0.395^**^	1	
Action (9)	15.776	3.410	0.304^**^	0.518^**^	0.376^**^	0.648^**^	0.112^**^	0.135^**^	0.454^**^	0.292^**^	1

^*^p < 0.05; ^**^p < 0.01.

#### 4.1.3 Measurement model validation

The measurement model was first evaluated before the mediation analysis. It was found that the latent constructs showed high convergent validity and acceptable internal consistency. Most of the factor loadings were higher than the recommended value, which confirmed that the observed items were adequate representations of the underlying latent variables. Construct three is not very satisfactory in terms of internal consistency with Attention (0.613), Interpretation (0.595), Responsibility (0.500), and Action (0.511), with an average variance extracted (AVE) of 0.50 or more. Although Knowledge (0.447) and Sense of Justice (0.395) had AVEs below the recommended threshold, their composite reliability (CR) remained acceptable.

Discriminant validity tests demonstrated that the square root of each latent variable, Average Variance Extracted (AVE), had a stronger association with the other measures, indicating that the measure is sufficiently differentiated. The multicollinearity diagnostics indicated that all variance inflation factors (VIFs) were less than 5, ranging from 1.20 to 2.87, thereby confirming the absence of multicollinearity issues.

#### 4.1.4 Mediating role test of physical self-efficacy and sense of justice

**Figure 2:**
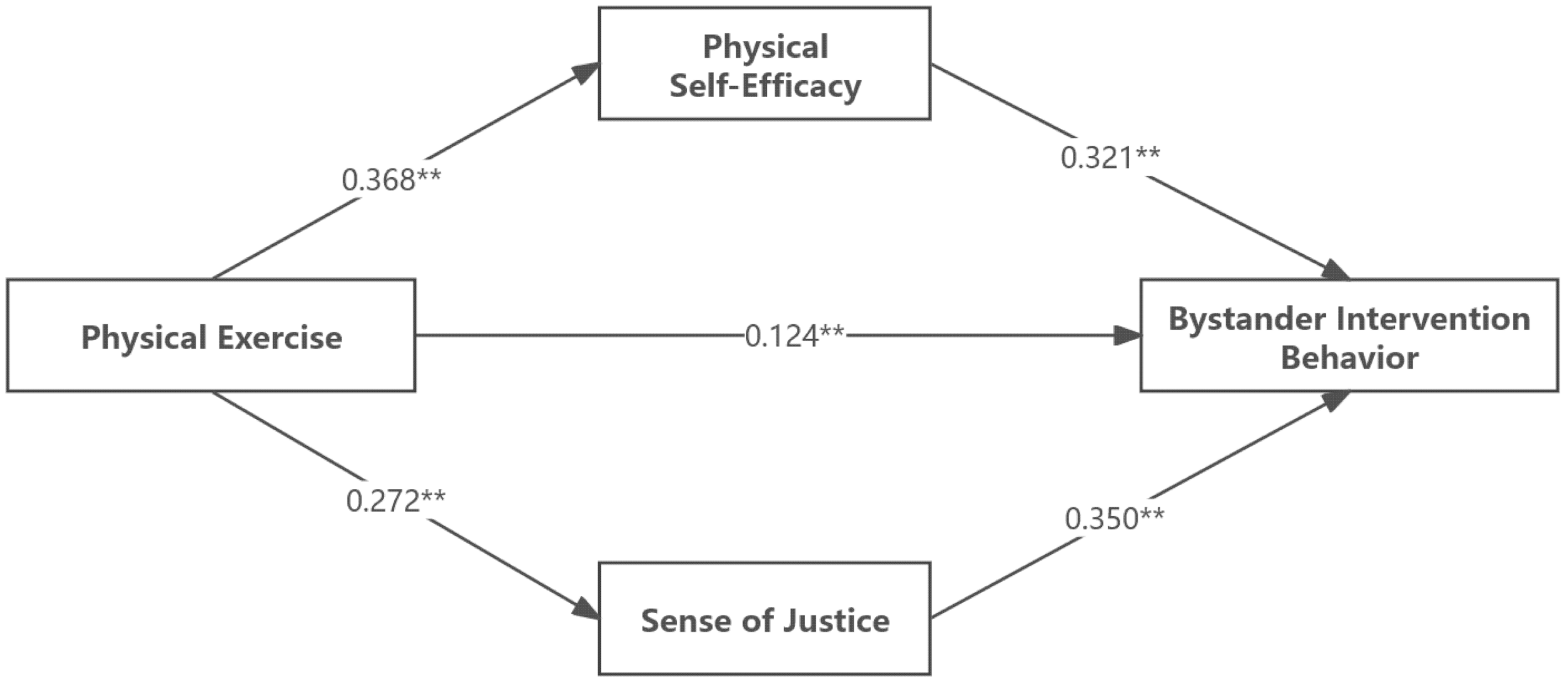
Path coefficients of the structural equation model of physical exercise on bystander intervention behavior in school bullying.

In this study, SPSS 27.0 and AMOS 28.0 were used to investigate the relationships between physical exercise, physical self-efficacy, sense of justice, and bystander intervention behavior in the context of school bullying. The results (Table 3 and Figure 2) suggest that physical exercise is correlated with bystander intervention behavior, considering the relevant factors (*β* = 0.124, *p* < 0.01). Both the physical self-efficacy and the perceptions of justice exhibited a positive and significant relationship with physical exercise, and both mediators were positively related to bystander intervention activity (*p* < 0.01). Both indirect effects, through physical self-efficacy (*β* = 0.118) and the feeling of justice (*β* = 0.095), turned out to be significant (*p* < 0.01).

**Table 3:** Mediating effects test of physical self-efficacy and sense of justice.

Explanatory variables		Explained variables			
		Intervening behavior (*β*)	Physical self-efficacy (*β*)	Sense of justice (*β*)	Intervening behavior (*β*)
Control variable	Sex	−0.023	−0.083^**^	−0.095	0.037
	Age	0.014	−0.059	−0.082	0.061
	Only child	−0.013	0.083^**^	−0.012	−0.036
	Grade	0.007	0.059	0.092	−0.044
Independent variable	Physical exercise	0.338^**^	0.368^**^	0.272^**^	0.124^**^
Intermediary variable	Physical self-efficacy				0.321^**^
	Sense of justice				0.350^**^
	*R* ^2^	0.116	0.165	0.094	0.365
	Align *R*^2^	0.112	0.161	0.089	0.360
	*F*	24.992^**^	37.504^**^	19.704^**^	77.609^**^

*β*, Beta; *R*^2^, Coefficient of determination; *F*, *F*-statistic; Significance levels: ^**^p < 0.01, ^*^p < 0.05.

The indirect effects were confirmed by the bootstrap study, which used 5,000 samples using the SPSS PROCESS MACRO. The overall effect of exercise on intervention behavior was 0.338. The confidence intervals of the indirect effects were 0.088–0.149 and 0.068–0.127, with the confidence intervals being statistically significant. The results indicate that the mediating variables are physical self-efficacy and the perception of justice, which account for 35.06 and 28.23% of the entire relationship, respectively.

#### 4.1.5 Effects of physical exercise on the five stages of bystander intervention behavior

**Figure 3:**
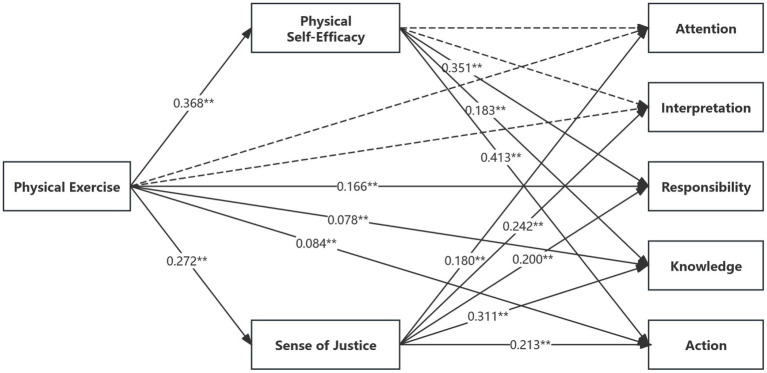
Path coefficients of the structural equation model for the process exercise of physical exercise on bystander intervention behavior in school bullying.

The AMOS structural equation modeling was carried out to examine the mechanisms through which physical exercise influences the five stages of bystander intervention (Table 4 and Figure 3). Model fit measures verified the order of the fit (*x*^2^/*df* = 2.418, RMSEA = 0.039, 90 percent CI = 0.036–0.041, SRMR = 0.039, CFI = 0.941, TLI = 0.936).

**Table 4:** Tests of mediation effects for the five steps of bystander intervention behavior.

Explanatory variables		Explained variables											
		Intervening behavior (*β*)					Physical self-efficacy (*β*)	Sense of justice (*β*)	Intervening behavior (*β*)				
		Attention	Interpretation	Responsibility	Know	Action			Attention	Interpretation	Responsibility	Know	Action
		Model 1	Mode2	Mode3	Mode4	Model 5	Model 6	Mode7	Model 8	Model 9	Model 10	Model 11	Model 12
Control variable	Sex	−0.038	0.015	−0.010	−0.006	−0.026	−0.083^**^	−0.095^**^	−0.016	0.040	0.038	0.039	0.029
	Age	0.030	0.001	−0.006	−0.043	0.012	−0.059	−0.082	0.048	0.022	0.031	−0.006	0.053
	Only child	−0.064^*^	−0.015	0.039	−0.082	0.065^*^	0.083^**^	−0.012	−0.066^*^	−0.015	0.012	−0.093^**^	0.034
	Grade	−0.037	−0.013	0.003	0.028	0.034	0.059	0.092	−0.057	−0.037	−0.036	−0.012	−0.009
Independent variable	Physical exercise	0.101^**^	0.123^**^	0.300^**^	0.230^**^	0.294^**^	0.368^**^	0.272^**^	0.032	0.046	0.116^**^	0.078^*^	0.084^**^
Intermediary variable	Physical self-efficacy								0.054	0.032	0.351^**^	0.183^**^	0.413^**^
	Sense of justice								0.180^**^	0.242^**^	0.200^**^	0.311^**^	0.213^**^
	*R* ^2^	0.016	0.015	0.094	0.058	0.099	0.165	0.094	0.052	0.072	0.265	0.199	0.322
	Adjusted *R*^2^	0.011	0.010	0.090	0.053	0.095	0.161	0.089	0.045	0.650	0.260	0.193	0.317
	*F*	3.034^**^	2.885^**^	19.782^**^	11.634^**^	20.897^**^	37.504^**^	19.704^**^	7.396^**^	10.512^**^	48.813^**^	33.604^**^	64.222^**^

There were no significant direct effects of physical exercise in the attention and interpretation stages (*p* = 0.359 and 0.185); however, it was fully mediated through the sense of justice (*p* = 0.049 and 0.066, 95% CI [0.027, 0.075]) and [0.043, 0.093], excluding 0. The feeling of justice was the overall impact in both phases, but physical self-efficacy did not have a significant effect.

Physical self-efficacy, as well as a sense of justice, partly mediated the relationship between the effects of physical exercise in the responsibility stage. Both physical self-efficacy and perception of justice showed positive but significant correlations with physical exercise, and neither mediator was positively related to bystander intervention behavior (*p* < 0.01). Physical self-efficacy stood out as the most significant mediating route (*β* = 0.129, *p* < 0.01, 95% CI [0.095, 0.164]) with a value of 43.10%. A sense of justice was also a secondary mediator (*β* = 0.054, *p* < 0.01, 95% CI [0.033, 0.082]) and contributed 18.12% of the effect.

Both the physical self-efficacy and sense of justice were partial mediators in the knowledge stage. The indirect effect through physical self-efficacy was 0.067 (95% confidence interval [0.038, 0.096]) and explained 29.26% of the total effect. Comparatively, the mediating effect of sense of justice (*β* = 0.085, 95% CI [0.057, 0.118]) was found to be more significant (36.78%), with a contribution of 36.78. These results indicate that the mediation pattern at this stage is dual, with indirect effects accounting for a significant proportion of the overall associations.

Physical exercises had an indirect relationship in the action stage, mediated by dual partial mediation through physical self-efficacy and sense of justice. The effect was significant in terms of direct effect (*β* = 0.413, *p* < 0.01) that contributed 51.61% of the overall effect and also in terms of indirect effect (*β* = 0.152, 95% CI [0.114, 0.192]) that contributed 51.70% to the overall effect. Even though the indirect influence of the sense of justice was still lower, it still had a substantial impact on the effect (20.73% accounted for by 95% CI [0.036, 0.084], *p* = 0.058). These findings demonstrate that self-efficacy played a leading role, and the sense of justice was a rank-two but irreplaceable factor.

### 4.2 Study 2: longitudinal intervention study

#### 4.2.1 Enhancing bystander intervention in school bullying through physical exercise

An 8-week longitudinal intervention, where the controlling variable was Sanda (martial arts), was conducted to investigate the correspondence of potential causation relationships among variables and the impact of exercise interventions on bystander action and underlying psychological mechanisms.

After the cross-sectional survey, 51 male students aged 12–16 who engaged in low physical activity (less than 1 extracurricular exercise session/week, less than 30 min/session) were recruited in School A in Nanchang. Only male students were recruited for the intervention study to reduce potential confounding effects of gender differences in physical performance, self-efficacy, and bystander behavior. This allowed for a more accurate assessment of the effects of Sanda training on the psychological mechanisms and bystander intervention behavior. To increase statistical power, future studies should consider expanding the sample size.

#### 4.2.2 Test subjects

After the cross-sectional survey, 51 male students aged 12–16 years, who were low in terms of exercise (less than 1 extracurricular activity session per week, with each session lasting less than 30 min), were recruited from School A in Nanchang. The research possesses sufficient statistical power to determine the observed effect, despite the tiny sample size. A post-hoc power analysis conducted on the group changes following the intervention indicated that the study possessed sufficient power. The results of the intervention study should be regarded as preliminary evidence of the potential influence of physical exercise on bystander behavior and related psychological processes. Subsequent research should use a larger sample size to enhance statistical power.

Male students were deliberately selected to reduce any confounding variables that could lead to gender discrepancies in physical performance, self-efficacy, and bystander behavior, facilitating a more accurate evaluation of intervention effects. Each group consisted of 23 and 28 responders, respectively. Participants in the experimental group were randomly allocated to this group, while the control group was assigned to the other group.

The inclusion criteria were: (1) Male students who were healthy and free from severe cardiovascular disease or other exercise contraindications; (2) Low levels of physical activity; (3) Written informed consent from both the student and the legal guardian. The eligibility criteria included: (1) prolonged membership in school sports teams or extracurricular athletic training; (2) documented severe psychological or behavioral issues; (3) prior experience or formal training in Sanda; and (4) 20% of total training sessions missed. The age and baseline physical activity levels were considered as the control variables in the further statistical analysis.

#### 4.2.3 Experimental design and intervention

A randomized controlled trial (RCT) design was employed, using an 8-week intervention period and three 90-min training sessions conducted weekly. The three outcome measures were assessed at three time points: pre-intervention (T0), mid-intervention (week 4; T1), and post-intervention (week 8; T2).

The experimental group was trained in Sanda in a structured manner, which included elements such as basic stances and footwork, punching, kicking, offensive and defensive combinations, and complete sparring exercises. The training was designed to enhance physical coordination, lower body stability, strength control, and teamwork skills. Certified physical education instructors conducted all sessions, and on-site supervision was implemented to ensure the safety of participants and fidelity to the intervention. A compliance rate of 80 per cent or more was regarded as a sign of compliance. The control group received no additional intervention regarding exercise, as they continued with their usual school activities.

#### 4.2.4 Measurement and data processing

In the cross-sectional study, the validation scale measured physical self-efficacy, perceived fairness, and bystander intervention behavior at T0, T1, and T2. The questionnaires were distributed and completed on-site, collected, and entered immediately. The entry of questionnaires was done by two researchers, who were not dependent on one another. All the data were anonymised to maintain the participants’ privacy.

To determine the variations between the experimental and control groups at three time points, an ANOVA was also performed as a repeated-measures ANOVA. The size of the effects (generalized eta-squared and partial η^2^) was calculated to establish the strength of the intervention effects.

#### 4.2.5 Test results

According to Mauchly’s evidence, none of the outcome variables followed the assumption of sphericity (*p*s < 0.01); therefore, Greenhouse–Geisser corrections were applied. A repeated-measures ANOVA revealed that time had significant main effects on physical self-efficacy, perceived fairness, and bystander intervention behavior (all *p* < 0.001), with general changes in all cases trending upward as the intervention progressed. Interaction Group-Time is also notable for each outcome (all *p* < 0.001), indicating that the change in time between the experimental and control groups was dissimilar.

With physical self-efficacy, the experimental group registered high levels of increment between the baseline (T0) and post-intervention (T2), and the effect size is substantial (*η*^2^*p* = 0.911). The significant effect of the group was determined, as it was found to have a significant effect (*p* = 0.031, *η*^2^*p* = 0.091), and the effect was positive, indicating that the experimental group improved more than the control group.

The perceived fairness and behavior of bystander intervention had significant effects over time (Time effects: *η*^2^*p* = 0.869 and 0.760, respectively). There were also significant Group × Time interactions (*η*^2^*p* = 0.859 and *η*^2^*p* = 0.771), indicating that the experimental group showed greater improvement at the end of the intervention (T2) compared to the control group. However, the group effect was not statistically significant in one of the perceived the perceived sense of justice (*p* = 0.166) and bystander intervention behavior (*p* = 0.079), indicating that there were no general group differences during the baseline.

**Figure 4:**
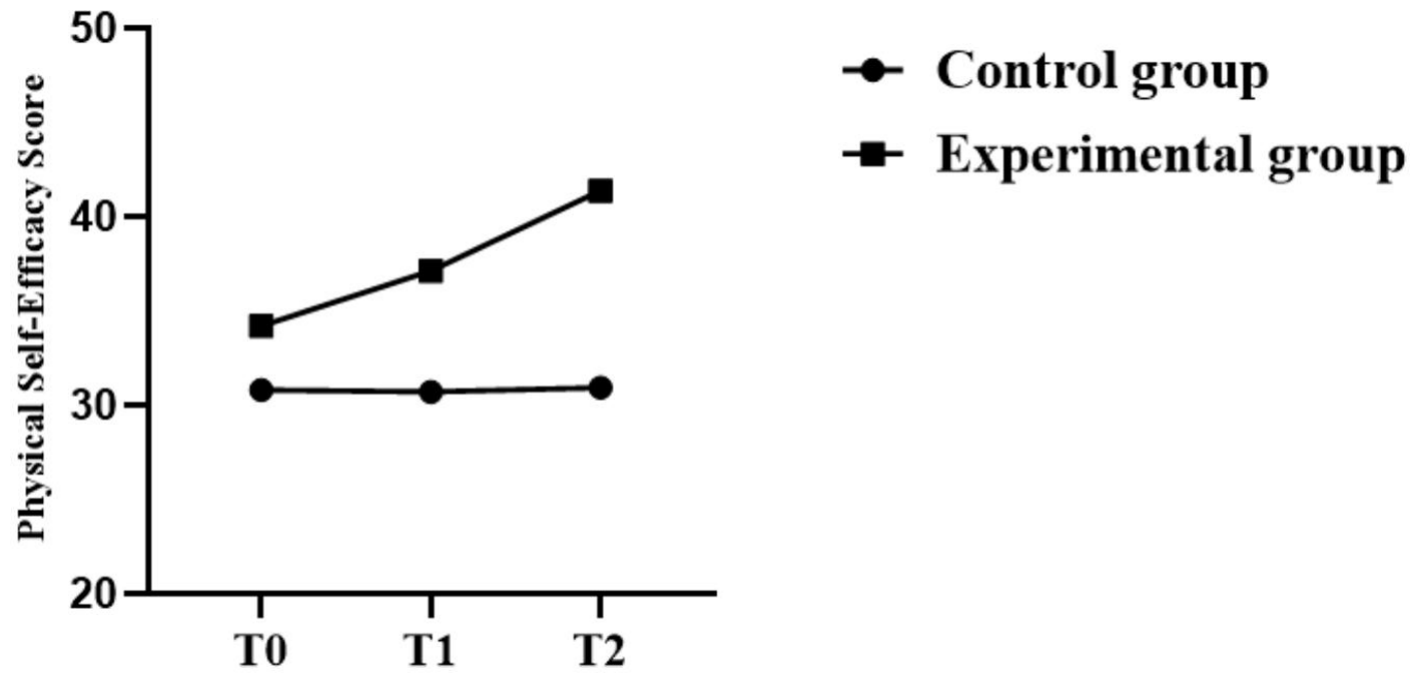
Trend chart of changes in physical self-efficacy.

**Figure 5:**
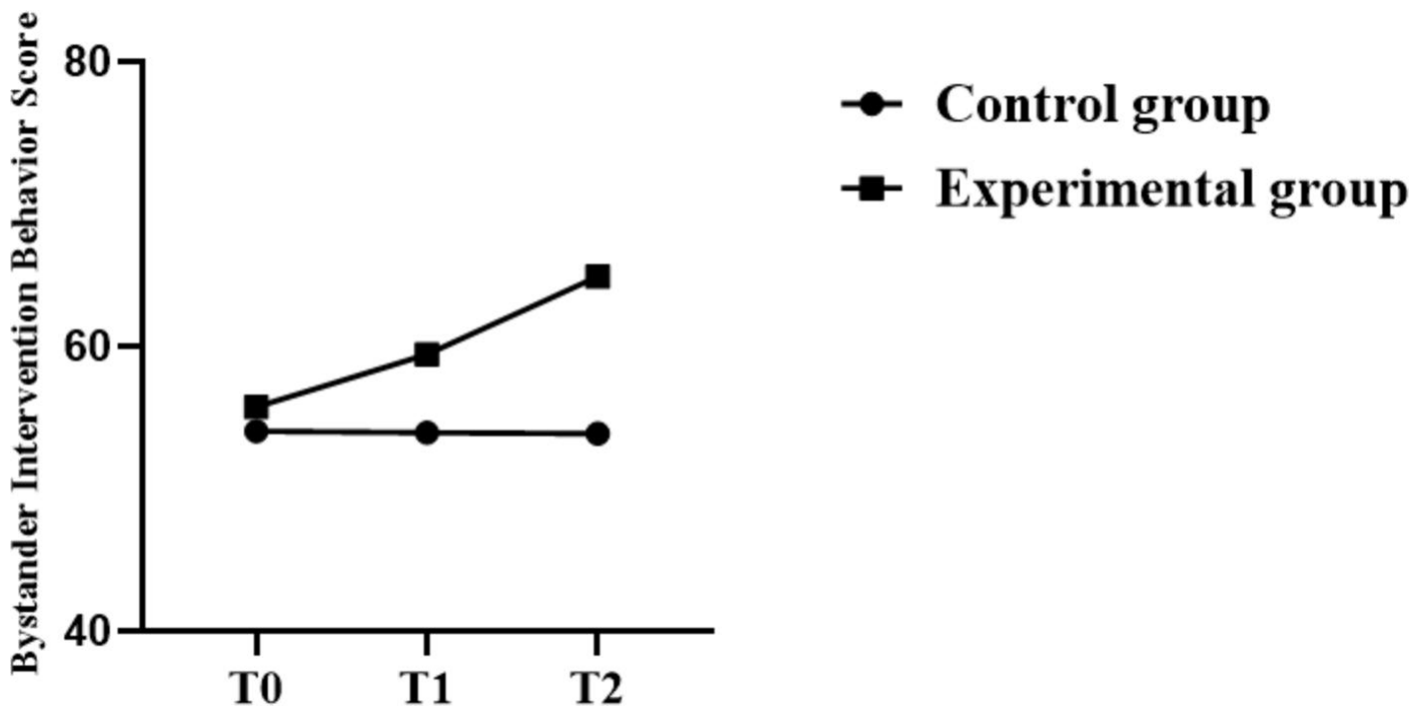
Trend chart of changes in sense of justice.

**Figure 6:**
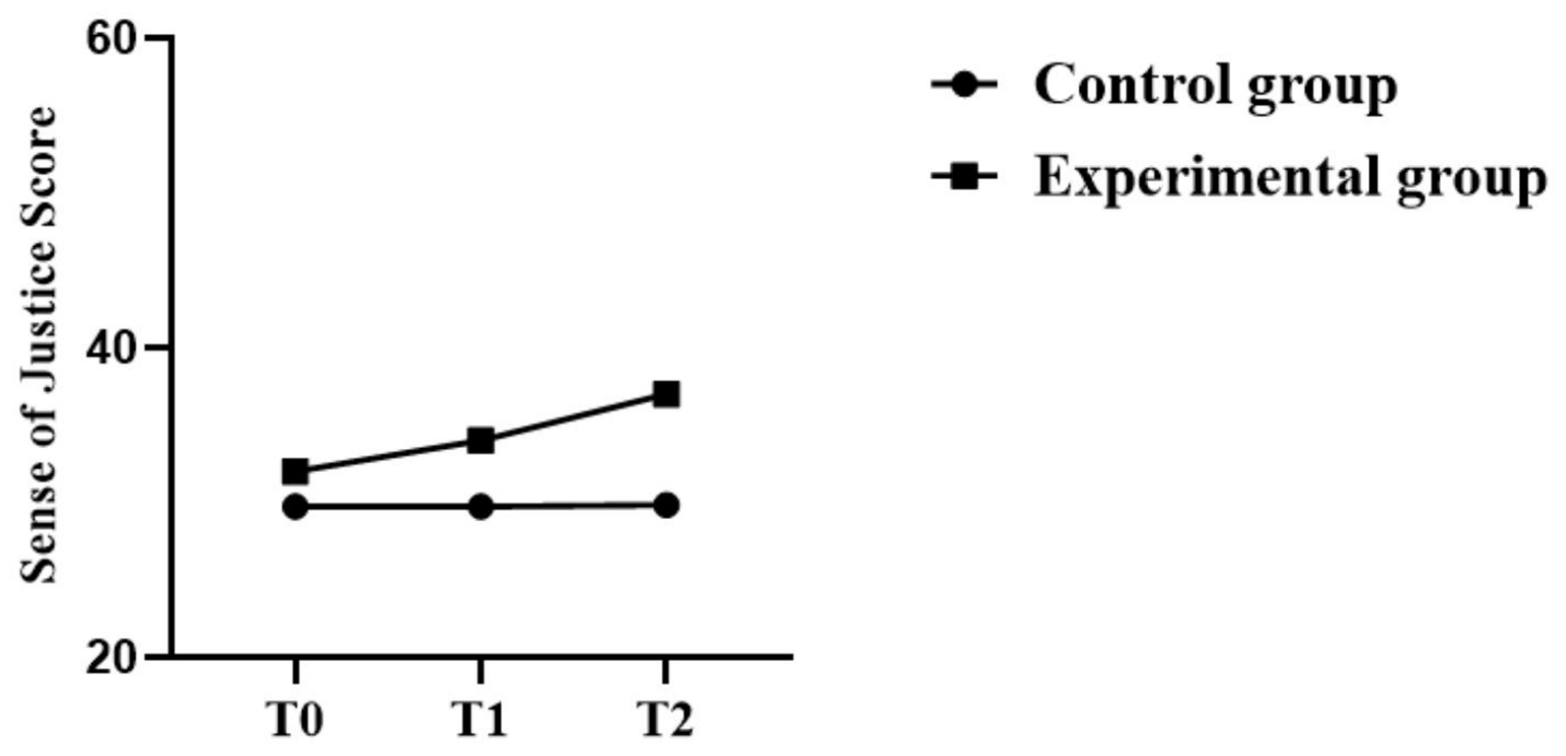
Trend chart of bystander intervention behavior.

All in all, the Repeated-Measures ANOVA revealed that the three outcome variables showed significant changes over time, and the change in each of the experimental groups was greater than that in the control group. The statistical outcomes and trends for the detained individuals are presented in Tables 5–7 and Figures 4–6.

**Table 5:** Results of repeated measures analysis of variance for physical self-efficacy after intervention.

Effect	Sum of squares SS (effect)	Sum of squares SS (error)	df (effect)	df (error)	Mean square (effect)	Mean square (error)	*F*	*p*	Generalized eta-squared	Partial *η*^2^
(Intercept)	177,240.762	17,268.466	1	49	177,240.762	352.418	502.928	<0.001	0.911	0.911
Group	1,730.253	17,268.466	1	49	1,730.253	352.418	4.910	0.031	0.091	0.091
Time	340.579	63.212	2	98	170.289	0.645	264.005	<0.001	0.019	0.843
Group: Time	317.520	63.212	2	98	158.760	0.645	246.131	<0.001	0.018	0.834

**Table 6:** Results of repeated measures analysis of variance for sense of justice after intervention.

Effect	Sum of squares SS (effect)	Sum of squares SS (error)	df (effect)	df (error)	Mean square (effect)	Mean square (error)	*F*	*p*	Generalized eta-squared	Partial *η*^2^
(Intercept)	155,743.909	19,394.810	1	49	155,743.909	395.812	393.479	<0.001	0.889	0.889
Group	783.439	19,394.810	1	49	783.439	395.812	1.979	0.016	0.039	0.039
Time	167.260	25.119	2	98	83.630	0.256	326.276	<0.001	0.009	0.869
Group: Time	152.829	25.119	2	98	76.414	0.256	298.125	<0.001	0.008	0.859

**Table 7:** Results of repeated measures analysis of variance for bystander intervention behavior after intervention.

Effect	Sum of squares SS (effect)	Sum of squares SS (error)	df (effect)	df (error)	Mean square (effect)	Mean square (error)	*F*	*p*	Generalized eta-squared	Partial *η*^2^
(Intercept)	492,260.943	21,245.502	1	49	492,260.943	433.582	1,135.336	<0.001	0.958	0.959
Group	1,393.335	21,245.502	1	49	1,393.335	433.582	3.214	0.079	0.061	0.062
Time	521.635	165.019	2	98	260.817	1.684	154.892	<0.001	0.024	0.760
Group: Time	554.733	165.019	2	98	277.367	1.684	164.720	<0.001	0.025	0.771

## 5 Discussion

### 5.1 Mechanisms of physical exercise’s influence on bystander intervention

The overall outcome of these findings is that there is some initial support for a dual-pathway model, which suggests that physical exercise can influence bystander intervention through both motivational-moral mechanisms and embodied behavioral preparedness. This framework models the net effects of physical and moral processes on bystander action, providing a generative framework for interpreting specific sub-mechanisms discussed below.

#### 5.1.1 Direct effects of physical exercise

The current study revealed that there was a positive relationship between bystander intervention and physical activity in the school bullying situation. A longitudinal observation revealed that students undergoing Sanda training exhibited an increase in intervention behaviors over time. These findings are in accordance with a social cognitive theory (Bandura, 1977, 1986, 1989; Bandura and Walters, 1977; Bandura and Wessels, 1997) according to which physical activity may affect decisions in two aspects: psychologically and behaviorally. The possible course of action of psychology is that exercise enhances the self-efficacy and perceived justice of bystanders, which prompts them to engage in helping behaviors. One such behavioral pathway is an increase in the capacity to act, which facilitates straightforward intervention in problem occurrences. In the context of embodied cognition (Liu, 2017; Baumeister et al., 2007; Kerzner, 2013), physical exercise can be used to apply the internal values to action through regular exercise and boost coping confidence.

This aligns with prior findings that motor skill acquisition and bodily practice can enhance moral sensitivity and prosocial behavior in adolescents (Ye, 2013), supporting the link between physical competence and social action.

Although these ideas are an initial step and grounded in observational data, they suggest that movement-based training may be used as a complement to traditional mental modalities in school bullying interventions, promoting more active helping skills. Study 2 provides initial evidence on the possible causal influences, whereby the growth of physical activity was related to an increase in intervention actions over time. However, cause-and-effect relations are to be understood with reservations.

Combined, these results suggest a dual-pathway model, whereby physical activity could affect bystander intervention by physically acting upon motivational/moral processes on the one hand and embodied behavioral preparedness on the other. This framework proposes an initial integrative approach to understanding how physical and moral processes contribute to bystander action, which can be further explored in subsequent sections through more specific processes, such as physical self-efficacy and sense of justice.

#### 5.1.2 Mediating role of physical self-efficacy

Our findings indicate that physical self-efficacy may moderate the connection between physical exercise and bystander intervention. In our longitudinal intervention study, students exhibited progressive enhancements in physical self-efficacy scores through participation in structured exercise programs, which correlated with improved bystander intervention behaviors. According to the domain-specific efficacy transfer theory proposed by Usher and Pajares (2008), Bandura and Wessels (1997), adolescents who believe they possess the necessary skills to address a specific problem are more inclined to take proactive measures in their interventions. Improvements in physiological regulation and perceptions of competence are utilized to enhance youngsters’ self-efficacy, thereby encouraging more proactive bystander behaviors (Liang, 1994; Driskell et al., 1994; Hepler and Andre, 2020; Kal et al., 2018).

This aligns with research indicating that elevated physical self-efficacy promotes proactive social engagement and protective behaviors in bullying scenarios (Guo et al., 2024). The same body action paradigm posits that elevated physical self-efficacy empowers adolescents to navigate evaluations and engage with societal intervention options more effectively. Such procedures can be beneficial during adolescence when body image and self-perception are vulnerable.

The findings suggest that exercise programs fostering physical self-efficacy can be enhanced by conventional anti-bullying techniques and the promotion of positive social ties. These are, however, provisional, and the causal links should be approached with caution.

This scenario pertains to the domain-specific transfer of efficacy, wherein confidence acquired in the physical realm can be applied to social interactions, as adolescents perceive it as a benchmark of achievement between their physical capabilities and the situational demands. Combined with the source of physical self-efficacy, this support appears to be one of the channels through which the motivational–moral dimension of the dual-pathway model can intervene, requiring action on the part of the bystander.

#### 5.1.3 Mediating role of sense of justice

These findings also suggest a moral pathway, whereby a sense of justice mediates the effect of physical exercise on interventional behavior. Short-term treatments failed to affect immediate overall perceptions of justice, yet gradual improvements occurred over time. In Study 2, participants in exercise interventions also developed a sense of justice over time, which was associated with increased bystander intervention. In line with Shields et al. (2005), feelings of fairness during physical exercise can inform moral judgements through embodied cognition. Sports may help internalize the value of such an idea, as righteous behavior is beneficial, and the positive feedback and utilization of physical experiences may trigger the intuitive moral reaction (Shao, 2020).

Similar results have been found linking team sports participation to enhanced fairness and moral reasoning in adolescents, highlighting the role of structured physical activity in moral development (Ludwiczak and Bronikowska, 2022). These cumulative experiences may enable people who witness such cases to respond more quickly to the situation. The results indicate that incorporating physical activity into school programs could positively influence the development of morality, as well as physical competence, and promote more active behaviors among bystanders. However, the interpretations are provisional because they are based on cross-sectional data and short-term longitudinal data.

In this respect, recurrent experiences of justice and compliance in exercising physical acts provide embodied grounds for intuitive moral reactions, as well as assist in the transition from passive viewing to active intercession. Therefore, the perception of justice is the moral-motivational aspect of the dual-pathway model that links physical self-efficacy to promote inclusive bystander responses.

### 5.2 Analysis of physical exercise, self-efficacy, and justice in bystander intervention

The inquiry analyzed the correlations among physical exercise, physical self-efficacy, and feelings of justice concerning the five stages of bystander intervention activity. Overall, physical activity and self-efficacy demonstrated the most substantial connections with subsequent levels of responsibility, knowledge, and action. A sense of justice was associated with all stages, particularly attention and interpretation. The results of this research indicate that some anti-bullying programs can be effective through the incorporation of physical, cognitive, and moral elements. In addition to the existing models of bystander intervention, the current study contributes to the body of knowledge by examining stage-specific associations. The results show that the effects of physical exercise, physical self-efficacy, and sense of justice on different stages of interventions are significantly different, thus providing empirical evidence for the two-pathway model presented in Section 6.1.

#### 5.2.1 The effect of physical exercise on the five stages of bystander intervention behavior

Physical exercise has shown that bystander intervention is linked to stage-specificity. The responsibility, awareness, and action phases contributed to positive results, while the attention and interpretation phases were minimally influenced. This trend supports the claim by Darley and Latané (1968) that different variables have different effects on the different intervention steps. The application of physical activity during the first stages can be primarily used to increase physical preparedness, self-efficacy, and emotional control (Bandura, 1977, 1989). During the responsibility phase, physical activities can enhance the belief that one can intervene and allow bystanders to be aware of possible actions.

During the knowing phase, exercise can be used to attain cognitive processing, which aids the bystanders in formulating effective intervention strategies as soon as possible. Physical preparedness developed as a result of exercise during the action stage leads to timely and accurate performance, decreasing hesitation and enhancing the quality of interventions (Zhou et al., 2024).

These stage-specific patterns align with prior findings showing that physical activity primarily supports later stages of social intervention where action readiness and cognitive control are crucial (Wan et al., 2021).

On the contrary, early attention and interpretations are based on ethical sensitivity and subjective judgment rather than on physical capability. Internalized moral schema is needed in order to recognize the cues of bullying, not embodied readiness; i.e., even people who are physically in good shape may not be able to recognize an interaction that is harmful to them when their moral sensitivity is low. Thus, although physical exercise may be used to support moral and cognitive elements of bystander intervention, it cannot be used in the judgment and awareness demanded in the earliest phases of the intervention.

This dual-pathway differentiation, based on physical readiness as supporting later-stage intervention and moral-cognitive processes as underpinning early-stage recognition and interpretation, is mutually supported.

#### 5.2.2 Stage-specific role of physical self-efficacy

Bystander intervention behavior was also found to have stage-specific relationships with physical self-efficacy. In analyzes of mediation, important effects were found in responsibility, knowing, and action levels, but none in attention and interpretation. The findings align with the triadic reciprocal framework proposed by Bandura (1977, 1989), which highlights the interplay among personal traits, behavior, and the situation. During the responsibility stage, physical self-efficacy facilitates bystanders in applying moral concern to perceived responsibility and assessing viable intervention actions. The higher an individual’s self-efficacy, the more likely they are to make an accurate assessment of their ability to act and determine effective options for interventions.

Self-efficacy is especially the case at the action stage. A high level of physical competence enhances confidence, improves neuromuscular coordination, and facilitates the prompt implementation of interventions (Zhi and Jian, 2014). In comparison, self-efficacy has little influence on attention and interpretation of the stages, which are predominantly based on moral cognition. It acts as a living engine that propels behavior after seeking action following the decision to take action with moral judgment. Nevertheless, it cannot substitute for the mental mechanisms involved in the perception and interpretation of bullying indicators. These findings imply that intervention programs must distinguish between the roles of moral cognition and physical self-efficacy, where the latter should help people take action, and the former should be involved in directing them during the initial assessment period.

This supports previous research indicating that physical self-efficacy translates moral motivation into concrete helping behaviors in contexts requiring immediate action, particularly in school bullying scenarios (Guo et al., 2024; Ryckman et al., 1982).

In this way, the behavior-embodied pathway of the dual-pathway framework, which facilitates the transfer of moral motivation into specific intervention behaviors in the late phases, can be regarded as physical self-efficacy.

#### 5.2.3 Influence of sense of justice across stages

In contrast to physical self-efficacy, the sense of justice had an impact on all five stages of bystander intervention, and some of the most significant effects were observed in the areas of attention and interpretation, where the effect of the sense of justice was fully mediated. Moral emotions, such as justice, are driven by what may be termed moral embodiment, which has been known to shape perception and reaction through embodied cognition. A sense of justice can promote an increased sensitivity to cues of bullying and empathy, allowing bystanders to identify ethical dilemmas and abusive relationships and interactions during the attention stage (Sun et al., 2014). Team sports play enhance the principles of fairness and protective actions, which increases the chances of bullying as an ethical concern.

The interpretation step involves directions in the process of justice (moral judgment) and the assignment of the position of bystanders to translate the abstract concept of justice into a tangible perception of the bullying phenomenon. Further involvement in the rules and norms of sports facilitates the identification of harmful practices and the assessment of whether these practices compromise the fairness factor. When performing the responsibility, knowing and acting stages, a sense of justice will be an incentive that compels the bystanders to go beyond the delegation of responsibility and into practical action. Moral courage also supplements physical self-efficacy, as it helps people to facilitate moral motivation in specific and assured interventions. Both justice and self-efficacy are dynamically connected: a person can act only with the help of physical ability, yet moral guidance helps to adhere to the morals and behave ethically.

This is consistent with literature indicating that structured physical activity promotes moral sensitivity across cognitive and action stages of bystander intervention (Ludwiczak and Bronikowska, 2022; Ju, 2013), highlighting the complementarity of moral cognition and embodied competence.

In general, the sense of justice is the motivational-cognitive route in a dual-pathways model, which serves as a guide throughout the pathway and constitutes a liaison of cognitive guidance, emotional arousal, and behavioral change. This perceptive network can obediently direct physical and moral education in the anti-bullying action plan.

## 6 Implications

This paper proposes that strenuous physical activity can be used as an effective supplementary measure to encourage bystander intervention in school bullying. Schools can employ various strategies to integrate group-based and individual exercise programs with existing anti-bullying initiatives and further develop moral consciousness, empathy, and social responsibility among students.

School mental health practitioners and teachers can partner to develop activities that enhance physical fitness while helping students develop prosocial skills, making them feel empowered enough to act in cases involving them. These results provide the basis for developing holistic intervention programs, i.e., combining cognitive-based education, social–emotional learning, and physical activity, which offer a viable model for decreasing bullying and improving the school climate.

## 7 Research limitations and prospects

The present study was a cross-sectional survey with a longitudinal intervention that looked at the impact of physical self-efficacy and sense of justice on bystander behavior toward school bullying. Although the creation of temporal precedence enhances causal inferences from the longitudinal intervention in Study 2, the results are preliminary evidence of causal relationships. Additionally, the cross-sectional nature of Study 1 does not permit firm conclusions regarding the causal direction.

Several constraints should be observed. First, all key variables were assessed through self-reported questionnaires, which may be subject to recall and social desirability biases. In Study 2, a small, all-male sample was used, and as such, the findings have limited generalizability. Self-reported bystander behaviors are not as validated as they could be due to the lack of direct behavioral observations or multi-informant data. Additionally, no behavioral or physiological manipulation checks were conducted to confirm improvements in physical competence after the intervention. Factors in untestable situations (e.g., school climate, teacher support, family engagement) and the cultural quirks of the school may also influence the results.

Further studies are needed to utilize larger and more heterogeneous samples, employ objective behavioral and physiological measures, adopt longer interventions, and investigate cross-cultural backgrounds. The inclusion of family and teachers, in addition to customized physical education programs, could contribute to the formation of multi-level bullying prevention approaches.

## 8 Conclusion

This paper suggests that adolescent bystander intervention in school bullying is positively related to physical exercise, and is associated with psychological factors such as confidence and moral motivation. The results indicate the significance of physical self-efficacy and sense of justice as central psychological mechanisms through which physical activity helps students become willing to take action in the event of observing bullying. The combination of evidence from the surveys and intervention makes the study a consistent description of using organized physical activity to stimulate proactive bystander response. These findings suggest that incorporating physical exercise into school-based bullying prevention can be a beneficial approach to fostering favorable peer conditions.

## Data Availability

The raw data supporting the conclusions of this article will be made available by the authors, without undue reservation.
